# Chromosome Aberrations in Lymphocytes of Patients Undergoing Radon Spa Therapy: An Explorative mFISH Study

**DOI:** 10.3390/ijerph182010757

**Published:** 2021-10-13

**Authors:** Nerea Paz, Carola Hartel, Elena Nasonova, Anna-Jasmina Donaubauer, Benjamin Frey, Sylvia Ritter

**Affiliations:** 1 Department of Biophysics, GSI Helmholtzzentrum fuer Schwerionenforschung, 64291 Darmstadt, Germany; nerea.paz@scsalud.es (N.P.); c.hartel@gsi.de (C.H.); nasonova@jinr.ru (E.N.); 2 Genetics Unit, Hospital Universitario Marqués de Valdecilla, 39008 Santander, Spain; 3 Laboratory of Radiation Biology, Joint Institute for Nuclear Research (JINR), 141980 Dubna, Russia; 4 Translational Radiobiology, Department of Radiation Oncology, Universitätsklinikum Erlangen, Friedrich-Alexander-Universität Erlangen-Nürnberg (FAU), 91054 Erlangen, Germany; Anna-Jasmina.Donaubauer@uk-erlangen.de (A.-J.D.); Benjamin.Frey@uk-erlangen.de (B.F.)

**Keywords:** radon, mFISH, clonal aberrations, complex aberrations, T-cell receptor genes

## Abstract

In the present exploratory study, we aim to elucidate the action of radon in vivo and to assess the possible health risks. Chromosome aberrations were analyzed in lymphocytes of two patients (P1, P2) undergoing radon spa therapy in Bad Steben (Germany). Both patients, suffering from painful chronic degenerative disorders of the spine and joints, received nine baths (1.2 kBq/L at 34 °C) over a 3-week period. Chromosome aberrations were analyzed before and 6, 12 and 30 weeks after the start of therapy using the high-resolution multiplex fluorescence in situ hybridization (mFISH) technique. For comparison, the lymphocytes from two healthy donors (HD1, HD2) were examined. P1 had a higher baseline aberration frequency than P2 and both healthy donors (5.3 ± 1.3 vs. 2.0 ± 0.8, 1.4 ± 0.3 and 1.1 ± 0.1 aberrations/100 analyzed metaphases, respectively). Complex aberrations, biomarkers of densely ionizing radiation, were found in P1, P2 and HD1. Neither the aberration frequency nor the fraction of complex aberrations increased after radon spa treatment, i.e., based on biological dosimetry, no increased health risk was found. It is worth noting that a detailed breakpoint analysis revealed potentially clonal aberrations in both patients. Altogether, our data show pronounced inter-individual differences with respect to the number and types of aberrations, complicating the risk analysis of low doses such as those received during radon therapy.

## 1. Introduction

Therapeutic exposure to the naturally occurring radioactive noble gas radon has beneficial effects for patients suffering from chronic painful degenerative diseases and inflammatory diseases of the musculoskeletal system [1,2,3,4]. During therapy, patients are repeatedly exposed to radon within a period of 3 to 4 weeks by bathing in radon-containing water (balneotherapy) or by visiting galleries or caves with a high natural radon concentration. After therapeutic radon application, patients report pain relief, an increase in joint mobility and a reduced consumption of analgesic drugs and these effects last for several months after treatment [2,5,6]. Yet, the mechanisms of action behind radon spa therapy are not fully understood [7]. 

Despite these positive effects of therapeutic radon exposure, it must be kept in mind that radon is radioactive and thus poses a health hazard. Out of the different existing isotopes, Rn-222 has the longest half-life (3.82 days) and decays by alpha particle emission [8]. A high concentration of indoor radon (chronic exposure) is associated with an elevated lung cancer risk [9,10]. Additionally, there is evidence for a relationship between radon concentration and the risk of leukemia [11]. The underlying cellular and molecular carcinogenic effects of radon have been reviewed by Robertson et al. [12]. For domestic indoor radon exposure as well as radon spa therapy, the radon activity has been reported, as it is readily measurable [11]. In contrast, the doses deposited in the lung and other organs cannot be measured directly and their calculations are affected by considerable uncertainties depending on the mechanism of radon intake (inhalation, ingestion, incorporation via the skin) and the distribution within the body [8,13,14,15]. 

An established method to assess the individual dose after (suspected) radiation exposure and to estimate the possible health risks (cancer risk) is the analysis of chromosome aberrations in the peripheral blood lymphocytes [16,17]. Presently, the dicentric chromosome assay is the gold standard for biological dosimetry, especially after a mass causality event [17,18]. However, standard staining techniques applied to detect dicentric chromosomes are unsuitable to uncover complex aberrations, which are a fingerprint of densely ionizing (high linear energy transfer, LET) radiation, including alpha particles (overview in [19]). To visualize this specific aberration type, a high-resolution staining technique such as multiplex fluorescence in situ hybridization (mFISH) has to be applied. As the mFISH method is much more time-consuming and expensive than the scoring of dicentric chromosomes, it is not suited for the investigation of a large number of individuals or for a high throughput analysis.

In the present exploratory study, we aim to gain a deeper insight into the cytogenetic effects of radon balneotherapy. Chromosomal aberrations were analyzed in peripheral blood lymphocytes of two patients undergoing therapy in Bad Steben, Germany, due to chronic degenerative disorders of the spine and joints. The radon baths (activity of 1.2 kBq/L) consisted of 9 immersions of 20 min each over a period of 3 weeks. In order to assess the possible health risks from radon treatment, blood samples were drawn from each patient before therapy and 6, 12 and 30 weeks after the start of therapy and were subjected to chromosome aberration analysis. For comparison, chromosome aberrations were analyzed in blood samples from two healthy volunteers with no known history of radiation exposure. To enable the detection of complex aberrations, the mFISH method was applied. To the best of our knowledge, this is the first time chromosome analysis in blood lymphocytes has been applied to investigate the health risks of radon spa therapy. These experiments are intended to build the basis for a cytogenetic study in a larger patient collective. 

## 2. Materials and Methods

### 2.1. Patients and Healthy Volunteers

Cytogenetic analysis was performed for two patients (P1, P2) that underwent radon spa therapy in the certified health resort Staatsbad Bad Steben (Germany). Patients suffered from degenerative spinal disorders with a pain intensity of ≥4 points on a visual analogue scale (persistent for at least one year) [20]. For comparison, two healthy donors (HD1, HD2) were used. The studies were conducted in accordance with the ethical principles of the Declaration of Helsinki. The patient study (also referred to as RAD-ON01 study, e.g., [20]) was approved by the ethical committee of the “Bayerische Landesaerztekammer” (vote reference: 12131, date of approval: 06.03.2013). Before participating in the study, patients signed an informed consent form. P1 was a 67-year-old female non-smoker, while P2 was a 50-year-old male former smoker. For the treatment, radon water at 1.2 kBq/L and 34 °C was used. Patients received 9 baths for 20 min each over 3 weeks [20]. P1 underwent radon spa therapy for the first time, for P2 it was the fifth therapy. Peripheral blood was drawn from each patient before as well as 6, 12 and 30 weeks after the start of therapy. Samples were transported to GSI (Darmstadt, Germany) and processed within 24 h after blood collection. The study involving healthy donors was approved by the institute’s international review board and informed consent was obtained from both volunteers. Healthy donor 1 (HD1) was a female non-smoker and provided five blood samples between the ages of 48 and 57. This data set has recently been published [21]. Healthy donor 2 (HD2) was a male aged 51 and a non-smoker and donated samples on two occasions within an interval of four months. For further analysis, data sets from the different blood donation times were pooled, as no significant differences were observed. 

### 2.2. Handling of Blood Samples 

Blood samples were drawn in BD Vacutainer^®^ CPT^TM^ tubes (Beckton Dickinson, Franklin Lakes, NJ, USA). Lymphocytes were isolated by centrifugation [22] and resuspended in an RPMI 1640 medium, supplemented with 20% fetal calf serum, 2 mM glutamine and 1% penicillin/streptomycin, referred to as a complete medium. All components were purchased from Biochrom (Berlin, Germany). Lymphocytes were seeded at a concentration of 0.5 × 10^6^/mL in a complete medium supplemented with 1% phytohemagglutinin (PHA, Life Technologies, Darmstadt, Germany) and 6–15 µg/mL 5′-bromo-2′ deoxyuridine (BrdU, Sigma-Aldrich, Germany). The cells were then incubated for 48 h at 37 °C, 5% CO_2_ and 95% humidity. To accumulate metaphases, 0.2 µg/mL colcemide (Roche, Mannheim, Germany) was added 3 h prior to harvest. 

### 2.3. Preparation and Staining of Chromosome Samples 

Chromosome samples were prepared and processed following the standard procedure [23]. Briefly, the cells were incubated with hypotonic solution (0.075 M KCl), fixed in 3:1 methanol: acetic acid, dropped on wet slides and air-dried. By means of the Fluorescence-Plus-Giemsa (FPG) technique, the percentage of metaphases in the first or a later cycle was determined as described elsewhere [24]. The analysis ensured that in all samples the proportion of first mitosis was higher than 90%. Other slides were subjected to mFISH, i.e., the slides were hybridized with a 24XCyte mFISH probe kit (MetaSystems, Altlussheim, Germany) according to the protocol recommended by the manufacturer. Metaphase search and automatic image acquisition were performed with a Zeiss Axioplan II microscope and Metafer4 software (Metasystems). Images were analyzed using Isis/mFISH software (MetaSystems). For further details, see [25]. To verify the presence of clonal aberrations, 2-color FISH was performed according to the manufacturer’s instructions (MetaSystems), staining the two chromosomes in question.

### 2.4. Aberration Scoring

For cytogenetic analysis, each chromosome of a spread was carefully examined and the chromosomes involved in an aberration were identified based on their unique fluorochrome profiles. Aberrations were classified following the mPAINT system [26] and grouped into the following categories: breaks (not associated with exchanges), simple exchanges and complex exchanges. Simple exchanges, originating from two breaks, typically comprise dicentrics, rings and translocations (including complete, incomplete and one-way forms). Notably, in the present study no ring chromosome was detected. Rearrangements involving three or more breaks in two or more chromosomes were classified as complex exchanges. The extent of complexity was determined using the CAB system, describing the minimum number of chromosomes (C), the number of arms (A) and the number of breaks (B) involved [26]. Complex aberrations were further grouped according to their potential transmissibility to daughter cells. They were classified as non-transmissible if they harbored a dicentric chromosome, a ring chromosome or an acentric fragment and classified as transmissible if they carried neither of those. When the occurrence of a clonal translocation was suspected, the breakpoints were localized by means of the ISIS software, i.e., the chromosome of interest was compared to a standard G-banded ideogram and the region where a break had occurred was determined. Translocations which appeared identical (within the resolution limits of the applied method) were termed “potentially clonal” when they were observed in two cells of an individual and were referred to as “clonal” when observed in three or more cells of an individual (in agreement with our previous work [27]). Details of the number of cells analyzed and the aberration types are given in Table 1. T-tests were used to determine statistical significance. 

## 3. Results

### 3.1. Chromosome Aberrations in Lymphocytes of Patients and Healthy Donors

**Figure 1:**
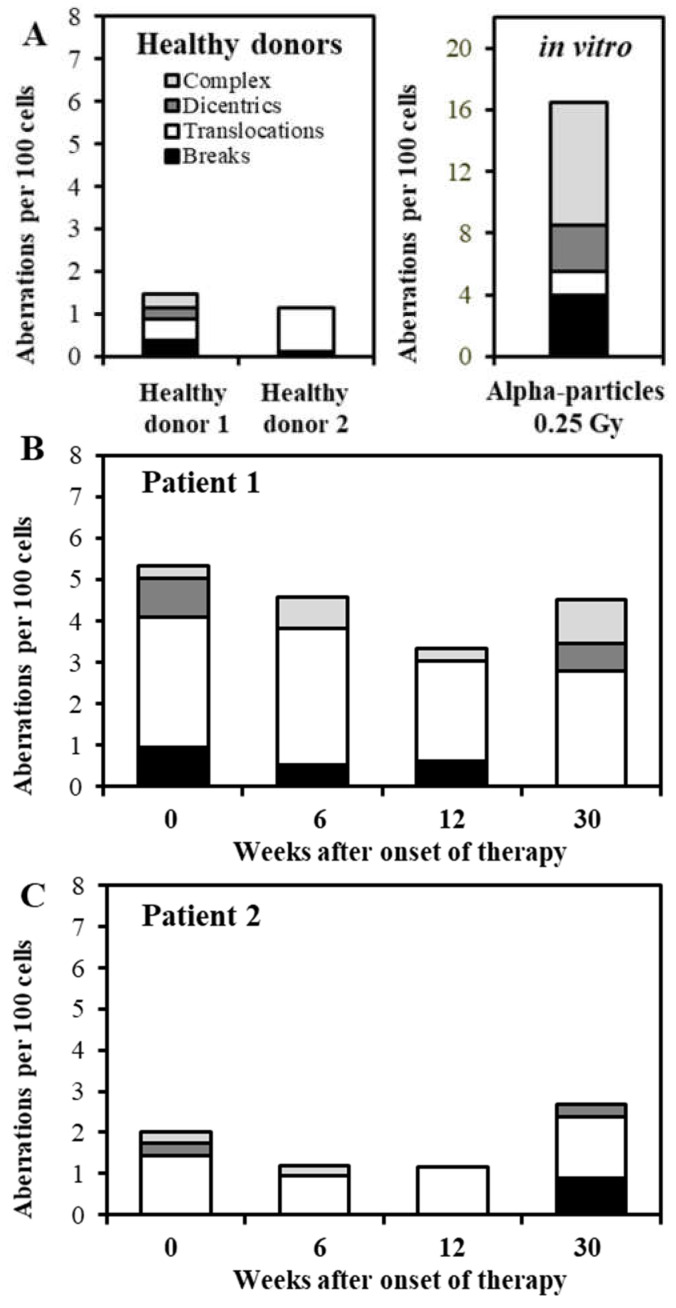
Aberration types (breaks, translocations, dicentrics and complex exchanges) detected by means of the mFISH technique. (**A**, left) shows the data for healthy donor 1 and 2, (**B**) for patient 1 and (**C**) for patient 2. For comparison, the data obtained after an in vitro exposure of lymphocytes from healthy donor 1 to 0.25 Gy alpha particles are displayed (**A**, right). Data for healthy donor 1 taken from [21].

Cytogenetic damage in peripheral blood lymphocytes of two patients was analyzed before and after radon spa therapy (Table 1). For P1 it was the first radon therapy, while for P2 it was the fifth treatment. For comparison, the aberrations were analyzed in the lymphocytes of two healthy donors (HD1, HD2, the data from HD1 have recently been published [21]). The frequencies of the different aberration types are shown in Figure 1 and Table 1 provides the absolute numbers of the different aberration types as well as the number of analyzed metaphases. Additionally, the data obtained for the lymphocytes of HD1 after in vitro irradiation with 0.25 Gy alpha particles (taken from [21]) are included. 

The baseline aberration frequency was similar for P2 and both healthy donors (*p* > 0.05), while it was significantly higher in P1 (*p* = 0.018). In all subjects studied, simple exchanges (translocations and dicentrics) represented the most frequent aberration category (>50% of all aberrations). The majority of simple exchanges were translocations; an elevated number of translocations was the main reason for the high number of aberrations detected in P1. The baseline frequency of the complex exchanges was low in the lymphocytes of HD1, P1 and P2 (0.003 per cell in each case), while no complex was observed in the lymphocytes of HD2. 

Finally, a comparison of the aberration types and frequencies before and after radon balneotherapy showed only small variations over time. Neither the aberration yield nor the frequency of complex aberrations increased significantly after therapy (*p* > 0.05).

### 3.2. Complex Aberrations

**Figure 2:**
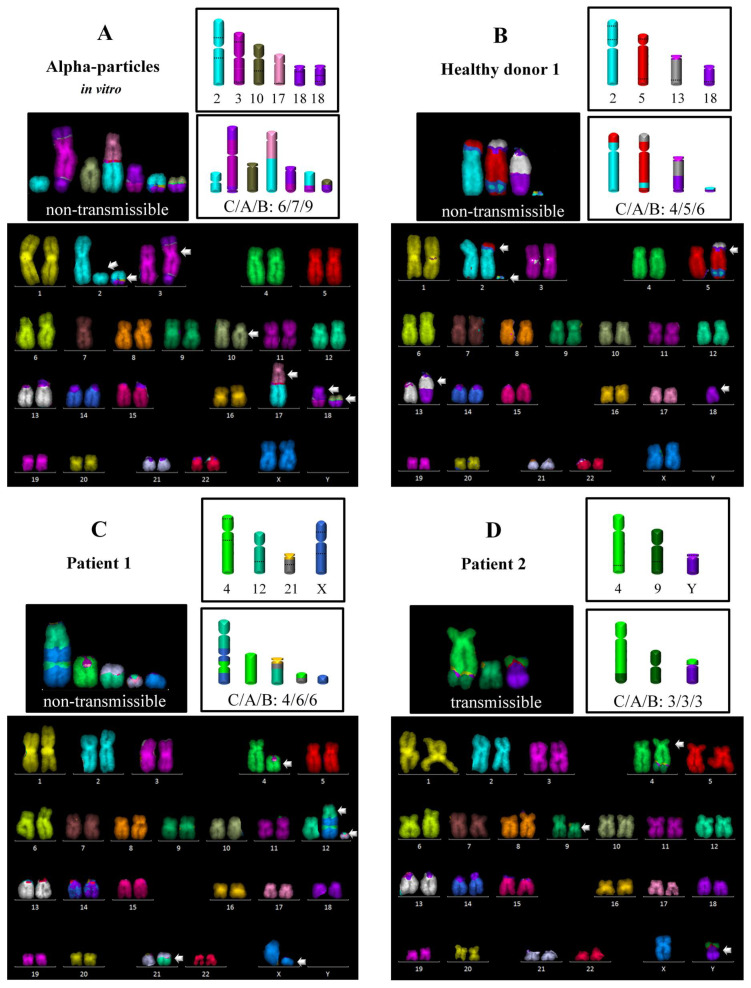
Representative karyotypes with complex aberrations revealed by the mFISH technique together with a schematic description of the breakpoints involved and the resulting rearrangements for cells of (**A**) healthy donor 1 exposed in vitro to 0.25 Gy alpha particles, (**B**) healthy donor 1 (control cells), (**C**) patient 1 (before therapy) and (**D**) patient 2 (six weeks after the onset of therapy). Additionally, the level of complexity is given according to the CAB system (number of chromosomes, chromosome arms and breaks involved). Arrows indicate the chromosomes involved in the respective complex rearrangement.

Next, we performed a detailed analysis of the complex exchanges observed in vivo and in vitro. Figure 2 shows examples of mFISH karyotypes with complex aberrations. As can be inferred from Table 1, in the lymphocytes of patients and healthy donors, the fraction of complex aberrations was low, ranging from 0 to 23% of all aberrations. This contrasts with the in vitro data, where complex exchanges represented the most frequent aberration type (48% of all aberrations). 

In Table 2, the complexity of each complex aberration in terms of the minimum number of chromosomes, arms and breaks involved (CAB) is given together with its potential transmissibility to daughter cells. Among the three subjects carrying complex aberrations, the complexity was highest in P1, involving on average 5.9 breaks in 3.6 chromosomes. Notably, 25% of the complex exchanges originated from >6 breaks. For HD1, the complexity was lower, involving a mean number of 4.3 breaks in 3.5 chromosomes and no complex with >6 breaks was found. The lowest level of complexity was observed for P2, who underwent the fifth radon spa therapy; both registered complexes originated from three breaks in two or three chromosomes.

We further determined the fate of each complex rearrangement (see Table 2). Non-transmissible forms represented the predominant type in HD1 (70%). In contrast, in P1 50% of the complexes were non-transmissible, while in P2 all the complexes were transmissible. Finally, a comparison of the pooled in vivo data from HD1, P1 and P2 with the in vitro data showed equal numbers of non-transmissible and transmissible aberrations in vivo (8:8), while the non-transmissible complex aberrations clearly dominated after in vitro alpha particle irradiation (15:1). 

### 3.3. Potentially Clonal Translocations 

To clarify whether some of the translocations observed were clonal in nature, we localized the breakpoints in all the translocated chromosomes detected in the patients and the healthy donors. Within the resolution limits of the method, this analysis provided evidence for the presence of potentially clonal aberrations in both patients (see superscripts in Table 1). To verify whether these translocations were clonal, 2-color FISH was performed. 

In the samples from P1, we found two cells carrying apparently the same translocation between chromosome 1 and 5 (one cell 6 weeks after the start of therapy, the other 30 weeks after therapy). However, the analysis of 600 additional metaphases using 2-color FISH for the chromosomes in question did not reveal any other copy of this aberration.

In P2 we detected three cells with a translocation between chromosome 7 and 14. In each cell, the breakpoint on chromosome 14 was located in band q11. In two cases (one cell before therapy, the other 6 weeks after the start of therapy), the breakpoint on chromosome 7 was mapped to band q34 indicating a potentially clonal origin. Yet, in the third cell (12 weeks after therapy), the breakpoint on chromosome 7 was different, i.e., mapped to band p13. These two distinct types of translocations between chromosome 7 and 14 have been described by others [28,29]. Consistent with the terminology of Dewald et al. [28] we refer in the following to t(7;14)(q34q11) as type I and to t(7;14)(p13;q11) as type II. The analysis of 1200 metaphases with 2-color FISH targeting chromosomes 7 and 14 revealed one additional type II translocation in P2, so that both types were classified as “potentially clonal”. It is also worth noting that we also detected a single lymphocyte with a type I translocation in P1 (6 weeks after the start of therapy) and in HD1.

## 4. Discussion

### 4.1. Cytogenetic Effects of Radon Spa Therapy 

Radon spa therapy has beneficial effects for patients suffering from chronic painful diseases [7]. However, as an alpha particle emitter, radon also poses a health risk. In this exploratory study, we analyzed chromosomal aberrations in peripheral blood lymphocytes of two patients undergoing radon spa therapy and, for comparison, in two health donors. 

During radon balneotherapy, the blood system plays a key role in the distribution of radon and its progeny throughout the body after intake via the skin from thermal water [30]. Moreover, the frequency of chromosomal aberrations in peripheral blood lymphocytes is a predictor of human cancer risk [16] and can be used for biodosimetry after a suspected exposure to ionizing radiation [17]. Application of the elaborate mFISH method allowed us to complete a detailed analysis of complex aberrations, which are considered as biomarkers for exposure to densely ionizing (high LET) radiation such as alpha particles [19]. This technique is highly sensitive as all human chromosome pairs are simultaneously labelled by 24 different combinations of fluorescent colors (e.g., [31]). 

In a recent experiment, where lymphocytes of a healthy donor (HD1) were exposed in vitro to alpha particles, we showed that complex aberrations were the most abundant aberration type induced [21], as was expected for high-LET radiation [19]. However, in the present study, no “fingerprint” for high-LET exposure was found in vivo. Complex aberrations were already present in both patients before the radon spa treatment as well as in one healthy donor and their frequencies did not increase after therapy. Likewise, the total aberration yield was not significantly increased by the radon spa therapy, giving no indication of an adverse health effect of radon spa therapy. This is in line with calculated dose estimations, which yielded a very low effective dose of radon balneotherapy (0.2–0.5 mSv [32]). 

In the present study, the mFISH method was chosen to fully reveal the pattern of complex aberrations. Yet, this technique is not well suited for the analysis of high numbers of metaphases. As a high number of analyzed metaphases is essential to detect small doses, in a subsequent study we are analyzing the cytogenetic effect of radon spa therapy in a larger patient cohort with the faster method of (semi-automatic) dicentric scoring, which is considered the gold standard for biological dosimetry [17]. 

### 4.2. Complex Aberrations

Complex aberrations were occasionally reported in lymphocytes of healthy donors [33,34] or astronauts before space missions [35] by means of 1–3-color FISH staining. We have previously described complex aberrations in a control sample of a healthy donor by means of the mFISH technique (i.e., HD1 [21,36]) but to the best of our knowledge, no other mFISH data on complex aberrations in lymphocytes of unexposed individuals have been published. When combining the data from unexposed samples (HD1, HD2 as well as P1 and P2 before therapy) a total of 8 complex aberrations were found in 3447 cells, i.e., 1 in 431 cells. Considering the fact that only in a few mFISH studies several hundred control cells were analyzed (because the technique is expensive and time-consuming), the lack of complex aberrations in the literature is not surprising [37,38,39]. 

Our in vitro experiments showed that complex aberrations were the most frequent aberration type induced by 0.25 Gy alpha particles, resulting on average from 6.4 breaks and only 6.25% were transmissible (1/16, Table 2), in line with data from Anderson et al. [40]. In contrast, in vivo (pooled data from patients + healthy donors), complex aberrations were less frequent and involved on average fewer breaks (5.3), but the fraction of transmissible complex aberrations was higher than in vitro, namely 50% (8/16, Table 2). In an in vivo mFISH study on the aberration frequency in the lymphocytes of workers with large body burdens of alpha-particle-emitting plutonium, Anderson et al. found that 33% of all complex aberrations were transmissible [41]. The high fraction of transmissible complex exchanges we observed in vivo (compared to chronic in vivo exposure and recent in vitro irradiation) hints at an accumulation of these aberrations over the lifespan, as is already well-known for translocations (i.e., simple transmissible exchanges) [42]. In contrast, a recent induction of complex aberrations would be characterized by predominantly non-transmissible complexes. Based on these considerations, it can be stated that the observed complex aberrations in the radon spa patients were apparently unrelated to the recent spa therapy.

### 4.3. Aberration Yield in Individuals

We found similar aberration yields in HD1, HD2 and P2, while they were significantly higher in P1. In both patients as well as in the normal donors, the aberration spectrum was dominated by translocations. Translocations are transmissible to daughter cells; they are known to accumulate with age in the lymphocytes of unexposed (control) subjects [42]. A comparison of our results with the reference database [42] showed the following number of translocations per 100 cells: P1 (67 years) 2.9 ± 0.5 vs. 1.3; P2 (50 years) 1.3 ± 0.3 vs. 0.83; HD1 (average 53 years) 0.49 ± 0.16 vs. 0.90; HD2 (51 years) 0.94 ± 0.31 vs. 0.85. Considering that the data were obtained using different methods (mFISH in our study vs. 1–3 color FISH in the database), this is a good agreement for HD1, HD2 and P2, while for P1 the yield of translocations was clearly higher than expected. We have no conclusive explanation for the cause of this increased aberration yield in P1, who underwent the first radon spa therapy and was a non-smoker (both in contrast with P2). Possible reasons for an elevated level of chromosomal aberrations are, for example, an exposure to ionizing radiation, antineoplastic drugs [43] (a study exclusion criterion was a malignant disease within the last five years [6]) or smoking habit (negated by P1). In patients suffering from chronic inflammatory diseases such as osteoarthritis or rheumatoid arthritis, chromosomal aberrations were found in cells from the synovial tissue but not in the lymphocytes [44]. Yet, to the best of our knowledge, no data for subjects suffering from chronic painful musculoskeletal diseases are available that can be used for comparison.

### 4.4. Clonal Aberrations

Clonal aberrations (in particular translocations) have been observed in the lymphocytes of occupationally or accidentally exposed individuals as well as in healthy (unexposed) subjects [31,45,46,47,48,49,50,51,52,53]. They arise from a single stem/progenitor cell that acquired the damage and passed it to its progeny. To examine whether clonally expanded translocations were present in the blood samples of healthy volunteers or patients, we localized the breakpoints of recurrent translocations. This analysis revealed two copies of t(1;5) in P1 and two copies of each t(7;14)(q32;q11) and t(7;14)(p13;q11) in P2. Thus, we found three potentially clonal translocations in P1 and P2 in total, but none in the healthy donors. As an aberration has to be multiplied during many cell divisions before it becomes visible in the peripheral blood as a clone, the observed potential clonal aberrations must have formed a long time ago and were therefore unrelated to the recent radon spa therapy.

It is worth noting that the two types of translocations between chromosomes 7 and 14 have previously been described and have been termed type I and II by Dewald et al. [28]. In fact, the occurrence of type I and type II translocations has been reported in PHA-stimulated lymphocytes of control individuals, patients and radiation workers [28,29]. Generally, the G-banding technique was applied for aberration detection. Based on these studies, the combined frequency of type I and type II translocations is about 5 × 10^−4^ per cell with type II slightly more frequent than type I (data are summarized in [29]). The finding that no type I or type II translocations were detected in metaphases of cultured fibroblasts, amniocytes or bone marrow cells suggests that they are T-cell specific, i.e., related to T-cell physiology [28]. This is further supported by the fact that T-cell antigen receptor genes are located at or near the particular breakpoints (T-cell receptor beta at 7q34; T-cell receptor gamma at 7p14; T-cell receptor alpha/delta at 14q11) [28,54].

To the best of our knowledge, there is no information on the frequency of type I and type II translocations in patients suffering from arthritic or musculoskeletal chronic painful degenerative diseases. Since in both patients type I and/or type II translocations were found and the combined frequency for P2 was higher than the average value (i.e., 2 × 10^−3^ per cell compared to 5 × 10^−4^ per cell [29]), these specific translocations might represent a disease-related phenomenon.

## 5. Conclusions

Radon spa therapy can have beneficial effects while radon also poses a health risk. In our exploratory mFISH study, we found no cytogenetic fingerprint of high-LET alpha particle radiation in the lymphocytes of two patients undergoing radon spa therapy. Complex aberrations, preferentially induced by alpha particles in vitro, were present in both patients, but their frequency was not increased compared to samples taken before radon spa treatment as well as samples from unexposed healthy donors. Moreover, the high fraction of transmissible complex aberrations in vivo was inconsistent with a recent formation by alpha particle exposure; instead, it indicates an accumulation of these aberrations during lifespan. Likewise, no effect of radon spa treatment on the aberration yield was found, providing no indication for a health risk posed by the radon balneotherapy. To validate these results, a subsequent study is underway with the application of a high throughput method (i.e., semiautomatic dicentric scoring) for the analysis of higher cell numbers and a larger patient group.

## Figures and Tables

**Table 1:** Number of aberrant lymphocytes and the corresponding aberration types visualized by the multiplex fluorescence in situ hybridization (mFISH) technique measured in healthy donors and in patients at the indicated times before and after the onset of therapy (6–30 weeks).

Subject	Sample	Cells Scored	Aberrant Cells (%)	Aberrations (Frequency)	Acentric Fragments (Frequency)	Translocations (Frequency)	Dicentrics (Frequency)	Complex Exchanges (Frequency)
Healthy donor 1 *	Control	1827	22 (1.2 ± 0.3)	26 (0.014 ± 0.003)	7 (0.004 ± 0.001)	8 ^b^ (0.004 ± 0.002)	5 (0.003 ± 0.001)	6 (0.003 ± 0.001)
Healthy donor 2	Control	957	11 (1.1 ± 0.3)	11 (0.011 ± 0.003)	1 (0.001 ± 0.001)	9 (0.009 ± 0.003)	0	0
Patient 1	Before	318	15 (4.7 ± 1.2)	17 (0.053 ± 0.013)	3 (0.009 ± 0.005)	10 (0.031 ± 0.010)	3 (0.009 ± 0.005)	1 (0.003 ± 0.003)
	6 weeks	394	18 (4.6 ± 1.1)	18 (0.046 ± 0.011)	2 (0.005 ± 0.004)	13 ^a,b^ (0.033 ± 0.010)	0	3 (0.008 ± 0.004)
	12 weeks	331	10 (3.0 ± 0.9)	11 (0.033 ± 0.010)	2 (0.006 ± 0.004)	8 (0.024 ± 0.008)	0	1 (0.003 ± 0.003)
	30 weeks	288	11 (3.8 ± 1.1)	13 (0.045 ± 0.012)	0	8 ^a^ (0.027 ± 0.010)	2 (0.007 ± 0.005)	3 (0.010 ± 0.006)
Patient 2	Before	345	6 (1.7 ± 0.7)	7 (0.020 ± 0.008)	0	5 ^b^ (0.014 ± 0.006)	1 (0.003 ± 0.003)	1 (0.003 ± 0.003)
	6 weeks	421	4 (0.9 ± 0.5)	5 (0.012 ± 0.005)	0	4 ^b^ (0.009 ± 0.005)	0	1 (0.002 ± 0.002)
	12 weeks	255	3 (1.2 ± 0.7)	3 (0.012 ± 0.007)	0	3 ^c^ (0.012 ± 0.007)	0	0
	30 weeks	334	9 (2.7 ± 0.9)	9 (0.027 ± 0.010)	3 (0.009 ± 0.005)	5 (0.015 ± 0.007)	1 (0.003 ± 0.003)	0

Superscripts indicate the presence of one cell carrying a specific reciprocal translocation (t). a: t (1;5), b: t (7;14)(q34;q11); c: t(7;14)(p13;q11). * Data taken from [21].

**Table 2:** Complex aberrations in lymphocytes of healthy donors and radon therapy patients visualized by mFISH. The minimum number of chromosomes (C), chromosome arms (A) and breaks (B) involved and the transmissibility (Y = yes, N = no) is given. For comparison, the data obtained for the lymphocytes of healthy donor 1 after in vitro exposure to 0.25 Gy ^241^Am alpha particles are added (taken from [21]).

	Complex	Complex Size	Trans-
	Nr	C/A/B	Missible
Healthy Donor 1	1	2/2/3	N
	2	2/2/3	Y
	3	3/3/3	Y
	4	4/5/6	N
	5	5/5/5	N
	6	5/5/6	N
Healthy Donor 2	0		
Patient 1	1	2/2/3	Y
	2	3/3/4	Y
	3	3/4/4	N
	4	4/4/5	Y
	5	4/4/5	N
	6*	4/6/6	N
	7	4/6/12	Y
	8	5/6/8	N
Patient 2	1 *	2/2/3	Y
	2	3/3/3	Y
Healthy Donor 1 0.25 Gy alpha- particles (in vitro)	1	2/2/3	N
	2	2/2/3	Y
	3	3/3/4	N
	4	3/3/4	N
	5	3/4/5	N
	6	3/4/6	N
	7	4/4/4	N
	8	4/4/5	N
	9	4/4/6	N
	10	5/5/7	N
	11	5/7/7	N
	12	5/7/11	N
	13	6/6/9	N
	14	6/6/9	N
	15	6/7/9	N
	16	7/8/11	N

* Aberration detected before radon spa therapy.

## Data Availability

Not applicable.
